# Alternative Strategies for Delivering Immunotherapeutics Targeting the PD-1/PD-L1 Immune Checkpoint in Cancer

**DOI:** 10.3390/pharmaceutics16091181

**Published:** 2024-09-07

**Authors:** Ryunosuke Hoshi, Kristyna A. Gorospe, Hagar I. Labouta, Taha Azad, Warren L. Lee, Kelsie L. Thu

**Affiliations:** 1 Laboratory Medicine and Pathobiology, Temerty Faculty of Medicine, University of Toronto, St. George Campus, Toronto, ON M5S 1A8, Canada; ryunosuke.hoshi@mail.utoronto.ca (R.H.); kristyna.gorospe@mail.utoronto.ca (K.A.G.); warren.lee@unityhealth.to (W.L.L.); 2 Keenan Research Centre for Biomedical Science, St. Michael’s Hospital, Toronto, ON M5B 1T8, Canada; hagar.labouta@unityhealth.to; 3 Leslie Dan Faculty of Pharmacy, University of Toronto, St. George Campus, Toronto, ON M5S 3M2, Canada; 4 Biomedical Engineering, Faculty of Applied Science and Engineering, University of Toronto, St. George Campus, Toronto, ON M5S 3E2, Canada; 5 Microbiology and Infectious Diseases, Faculty of Medicine and Health Sciences, Université de Sherbrooke, Health Campus, Sherbrooke, QC J1K 2R1, Canada; taha.azad@usherbrooke.ca; 6 Research Center, Centre Hospitalier Universitaire de Sherbrooke (CHUS), Sherbrooke, QC J1J 3H5, Canada; 7 Biochemistry, Temerty Faculty of Medicine, University of Toronto, St. George Campus, Toronto, ON M5S 1A8, Canada; 8 Medicine and the Interdepartmental Division of Critical Care Medicine, Temerty Faculty of Medicine, University of Toronto, St. George Campus, Toronto, ON M5B 1T8, Canada

**Keywords:** solid tumors, PD-1/PD-L1 immune checkpoint, immunotherapy, immune checkpoint inhibitors, irAEs, locoregional drug delivery, oncolytic virus, nanoparticle, ultrasound and microbubbles

## Abstract

The programmed death-1/programmed death-ligand 1 (PD-1/PD-L1) immune checkpoint constitutes an inhibitory pathway best known for its regulation of cluster of differentiation 8 (CD8)^+^ T cell-mediated immune responses. Engagement of PD-L1 with PD-1 expressed on CD8^+^ T cells activates downstream signaling pathways that culminate in T cell exhaustion and/or apoptosis. Physiologically, these immunosuppressive effects exist to prevent autoimmunity, but cancer cells exploit this pathway by overexpressing PD-L1 to facilitate immune escape. Intravenously (IV) administered immune checkpoint inhibitors (ICIs) that block the interaction between PD-1/PD-L1 have achieved great success in reversing T cell exhaustion and promoting tumor regression in various malignancies. However, these ICIs can cause immune-related adverse events (irAEs) due to off-tumor toxicities which limits their therapeutic potential. Therefore, considerable effort has been channeled into exploring alternative delivery strategies that enhance tumor-directed delivery of PD-1/PD-L1 ICIs and reduce irAEs. Here, we briefly describe PD-1/PD-L1-targeted cancer immunotherapy and associated irAEs. We then provide a detailed review of alternative delivery approaches, including locoregional (LDD)-, oncolytic virus (OV)-, nanoparticle (NP)-, and ultrasound and microbubble (USMB)-mediated delivery that are currently under investigation for enhancing tumor-specific delivery to minimize toxic off-tumor effects. We conclude with a commentary on key challenges associated with these delivery methods and potential strategies to mitigate them.

## 1. Introduction

Cancer encompasses a pathologically heterogeneous cluster of diseases defined by uncontrolled cell proliferation and the ability to metastasize throughout the body [1]. They are broadly categorized as either solid or hematologic malignancies [2], and both arise from the accumulation of genetic and epigenetic aberrations as a result of germline mutations, aging, carcinogen exposure, and chronic inflammation [3,4]. Cancer continues to impose a worldwide health burden, with an estimated 20 million new cases and 9.7 million deaths in 2022 [5]. While the incidence of various cancers varies by region, lung cancer is the most frequently diagnosed cancer worldwide, followed by breast, colorectal, prostate, and stomach cancers [5]. Lung cancer is also the leading cause of cancer-related mortality and is responsible for more deaths than colorectal, liver, breast, and stomach cancers combined [5]. Alarmingly, the global cancer burden is projected to increase to 28.4 million cases by 2040 [6], emphasizing the need for improved prevention, detection, and therapeutic strategies.

Traditionally, surgery has been a mainstay treatment for localized solid tumors [7]. Contemporary surgical methods including robotic and laparoscopic procedures are highly precise and minimally invasive [8], offering a potential cure for many early-stage cancers. However, surgery is unlikely to be a curative solution for patients with solid tumors that are diagnosed at advanced stages [9]. While they may receive surgery as palliative care, most patients with advanced cancer are treated with radiation, chemotherapy, immunotherapy, and/or molecular-targeted therapies that inhibit specific oncogenic driver mutations [10]. Although these treatment modalities often achieve robust anti-tumor responses that prolong the survival times of patients [11], their efficacy can be encumbered by intrinsic or acquired resistance [12,13,14,15], which are driven by genomic instability [16], intra-tumor heterogeneity [17], and suppression of anti-tumor immunity stimulated by therapy [18,19]. These common features of cancer enable the acquisition of genetic alterations that activate compensatory signaling pathways, clonal expansion of treatment-refractory cancer cell populations, and escape from immunosurveillance despite ongoing treatment interventions, respectively [13,20,21,22,23]. As a result, the therapeutic benefits of these conventional treatments are often ephemeral, and initial tumor regressions are followed by disease relapse and tumor progression. Another significant challenge that limits the efficacy of these treatment modalities is the manifestation of adverse side effects because of their incomplete tumor selectivity [24,25,26]. Ionizing radiation inadvertently damages healthy tissues surrounding the tumor [26], chemotherapy is frequently associated with systemic toxicities as their mode of action is rarely tumor-specific [27], and immunotherapies can promote overactivation of the immune system, compromising healthy organs [28]. Even targeted therapies that inhibit signaling pathways deregulated by driver oncogenes are not exempt from adverse toxicities because these pathways also have homeostatic roles in non-malignant cells [29]. Such off-tumor toxicities often require treatment termination in patients whose tumors are responding to therapy, which unfortunately limits tumor control and also contributes to disease progression. Thus, new strategies to focus the therapeutic effects of standard-of-care treatments preferentially to tumor tissues are needed to increase their efficacy and minimize toxicity. In this review, we focus specifically on alternative approaches to administering immunotherapies to limit their adverse off-tumor effects.

## 2. Cancer Immunotherapy

### 2.1. Reversing Tumor Immune Evasion with Immune Checkpoint Inhibitors

Immunotherapies can elicit durable responses of ten years or more in some patients and have revolutionized the cancer treatment landscape [30]. Evasion of immune destruction is a major hallmark of cancer [31], and tumors use a variety of immunosuppressive mechanisms including loss of neoantigen expression; downregulation of antigen presentation; recruitment of immunosuppressive immune cells such as regulatory T cells [32,33], myeloid-derived suppressor cells (MDSCs), and CD71^+^ erythroid cells [34,35]; and upregulation or secretion of immune-modulatory molecules such as anti-inflammatory cytokines and inhibitory checkpoint proteins (e.g., PD-L1) to accomplish this feat [36]. These mechanisms effectively disrupt the cancer immunity cycle—a series of events starting with the release and detection of tumor antigens by professional antigen-presenting cells (APCs) and terminating with cancer cell elimination [37,38]. Tumors acquire the ability to escape cancer immunity by undergoing a process known as immunoediting [39] which consists of three sequential steps: elimination, equilibrium, and escape. The elimination phase involves the recognition and engulfment of cancer cells by reconnoitering APCs in a process called immunosurveillance, leading to the activation of effector T cells and induction of tumor cell killing. In the equilibrium phase, the immune system and cancer cells are in a stalemate where the tumor is contained but not eliminated entirely. In the final escape phase, selective pressure exerted by the immune system promotes the survival and proliferation of cancer cells capable of evading immune destruction, resulting in disease progression [38,39]. Immunotherapies aim to reverse this process by directly activating immune cells and/or altering immunomodulatory signaling pathways [40].

**Figure 1:**
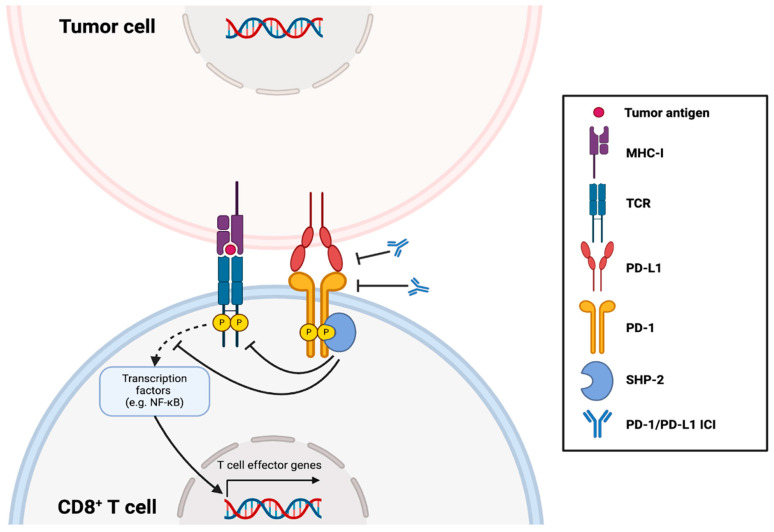
PD-1/PD-L1 Signaling. Engagement of PD-L1 with the PD-1 receptor on activated T cells triggers the recruitment of SHP-2 to the cytoplasmic tail of PD-1, which inhibits proximal TCR signaling necessary to induce T cell effector genes. PD-1/PD-L1 ICIs block this inhibitory signaling pathway to disinhibit T cell functionality.

Immunotherapeutic agents used in the clinic include cytokines, adoptive cell therapy, cancer vaccines, and immune checkpoint inhibitors (ICIs) [40,41]. Relevant to this review, immune checkpoints are physiological gatekeepers of innate and adaptive immune responses and are governed by receptor-ligand pairs that regulate the dynamic interplay between immunopermissive and immunosuppressive signals [42]. Checkpoints can either activate or inhibit immune responses and proper integration of polarizing immune signals is paramount for immune homeostasis. While stimulatory immune checkpoints provide protection from pathogens and malignancy, their overactivation can damage healthy tissues. Inhibitory checkpoints exist to maintain self-tolerance and prevent autoimmunity [43]; as such, many cancers upregulate inhibitory immune checkpoints to avoid immune-mediated clearance [44]. Discoveries of tumors exploiting this immunosuppressive mechanism have stimulated the development of inhibitors to antagonize inhibitory immune checkpoints, including those targeting T cell immunoglobulin and mucin domain-containing protein 3 (TIM-3) [45], lymphocyte activation gene 3 (LAG-3) [46], cluster of differentiation 47 (CD47) [47], cytotoxic T-lymphocyte-associated antigen 4 (CTLA-4) [48], and programmed death-1/programmed death-ligand 1 (PD-1/PD-L1) [49]. This review focuses on ICIs targeting the PD-1/PD-L1 signaling axis that suppresses T cell-mediated immunity (Figure 1) [50], which we will refer to as PD-1/PD-L1 ICIs. These ICIs have become the most widely used cancer immunotherapies owing to their approval by the United States Food and Drug Administration (U.S. FDA) starting in 2014 for PD-1 ICIs and 2016 for PD-L1 ICIs, and their demonstrated efficacy in a variety of cancers [51,52]. Below we describe the PD-1/PD-L1 checkpoint, PD-1/PD-L1 ICIs, and clinical limitations that should be addressed to maximize their potential to benefit cancer patients.

### 2.2. PD-1/PD-L1-Targeted ICIs

PD-1 is a cell-surface receptor present on many immune cells including natural killer (NK) cells, macrophages, and dendritic cells (DCs), but most prominently on T cells upon their activation [53,54]. In response to an infection, transcription factors such as fork-head box protein O1 (FOXO1) and nuclear factor of activated T cells, cytoplasmic 1 (NFATC1) upregulate the expression of PD-1 on antigen-activated T cells as they eliminate the pathogen [55]. On the other hand, its ligand PD-L1 is constitutively expressed in various cells throughout the body [55]. Physiologically, PD-1/PD-L1 signaling sustains tolerance during adaptive immune responses by suppressing the overactivation of effector T cells which could otherwise lead to harmful autoimmunity. At the molecular level, engagement of PD-1^+^ T cells with PD-L1^+^ cells results in phosphorylation of the cytoplasmic tail of PD-1, leading to the recruitment of SH2 domain-containing tyrosine phosphatase (SHP-2). SHP-2 then attenuates downstream T-cell receptor (TCR) signaling which diminishes effector gene expression and increases apoptotic gene expression, inactivating or killing the T cells to de-escalate effector responses [56].

Usually, PD-1 levels are downregulated upon pathogen clearance, but in the event of continuous antigen stimulation during chronic infections, PD-1 levels on effector T cells remain elevated. This eventually creates a population of T cells termed ‘exhausted T cells’ [57]. Terminally exhausted T cells with high levels of PD-1 are, by phenotypic definition, dysfunctional. They become incapable of interleukin-2 (IL-2), tumor necrosis factor-α (TNF-α), interferon-γ (IFN-γ), and granzyme B (GzmB) synthesis, all of which dampens their inflammatory and cytotoxic functions [58]. Analogous to chronic infections, tumor-infiltrating T cells in the tumor microenvironment (TME) are continuously exposed to tumor antigens, transforming them into exhausted, hyporesponsive states through PD-1 upregulation [58,59]. Furthermore, overexpression of PD-L1 in many cancers, either on tumor cells themselves or others such as APCs, exacerbates the immunosuppressive nature of the TME [58]. Studies have shown that PD-L1 is upregulated, at least in part, by the secretion of IFN-γ by activated, functional T cells [60], which enhances nuclear factor kappa B (NF-κB)-driven expression of PD-L1 in tumors [53]. Although distilled to its skeletal framework, expression of PD-1 on T cells and PD-L1 in the TME facilitates inhibitory signaling that effectively counteracts adaptive tumor immunity to enable tumor immune escape. Thorough descriptions of signaling driven by PD-1/PD-L1, their transcriptional regulation, and their roles in immune evasion can be found in recent reviews [53,55,61,62].

The goal of PD-1/PD-L1 ICIs is to block the interaction between PD-1 and PD-L1 to inhibit PD-1 signaling, reverse T cell exhaustion, and re-activate anti-tumor immune responses to promote tumor regression. Clinically approved PD-1/PD-L1 ICIs are intravenously (IV) administered monoclonal antibodies (mAb) targeting either PD-1 or PD-L1 that effectively block their ligation to abrogate inhibitory signaling [63]. Ample clinical evidence has shown that PD-1/PD-L1 ICIs can revive T cell function and extend progression-free and overall survival of cancer patients [64,65] even in individuals with advanced disease [66,67]. Since they demonstrate anti-tumor efficacy that is superior to standard chemotherapies in patients with various malignancies [67,68], PD-1/PD-L1 ICIs are now first- and second-line treatments for 19 different malignancies [69]. To date, six ICIs targeting PD-1 (pembrolizumab, nivolumab, cemiplimab, retifanlimab, dostarlimab, and toripalimab), and three targeting PD-L1 (atezolizumab, avelumab, and durvalumab) have been approved [70]. Treatment with PD-1/PD-L1 ICIs can be prescribed based on tumor biomarkers including PD-L1 expression, tumor mutation burden (TMB), CD8^+^ T cell infiltration, microsatellite instability (MSI) [71], and TCR clonality [72]. Effective treatment regimens include monotherapy, as well as combination or sequential therapy with other ICIs, targeted therapy, or chemotherapy [69,73,74]. Given the ability of PD-1/PD-L1 ICIs to elicit durable tumor regressions in some patients, further research is warranted to better understand how such responses could be harnessed to benefit more patients, and how to mitigate some of their efficacy-restricting limitations as described below.

### 2.3. Limitations of PD-1/PD-L1 Blockade

Not all cancer patients are eligible for treatment with PD-1/PD-L1 ICIs, and in those who are, objective responses are not ubiquitous [75]. Several factors contribute to suboptimal anti-tumor responses. First, the biomarkers used to predict response to PD-1/PD-L1 ICIs, particularly tumor expression of PD-L1, are imperfect [62]. Some patients whose tumors exhibit high PD-L1 expression are completely insensitive to PD-L1 blockade [76], whereas others with PD-L1-negative tumors respond [77]. This emphasizes the need to refine the current criteria used to select patients for PD-1/PD-L1 ICI therapy. Interestingly, accumulating preclinical and clinical evidence indicates that several variants of the PD-1/PD-L1 proteins can be expressed and detected in tumors as a result of post-translational modifications (PTMs) [78], single nucleotide polymorphisms (SNPs) [78,79,80], and alternative splicing events [81]. As proposed for alternatively spliced isoforms of PD-L1 in melanoma [81], such variants could influence the efficacy of ICIs if they prevent ICI binding to PD-1/PD-L1, consequently diminishing their therapeutic effects. Therefore, such variants may emerge as response-predictive biomarkers to inform patient selection for PD-1/PD-L1 ICI therapy.

Second, intrinsic and acquired resistance limit response rates and duration. Intrinsic resistance, defined as a lack of therapeutic response, can be attributed to multifactorial resistance mechanisms including an immunologically ‘cold’ immunosuppressive TME [82], suppression of PD-L1 expression [83], and exclusion of T cells from the TME [84]. On the other hand, acquired resistance following an initial response can arise through various mechanisms including upregulation of other inhibitory checkpoints such as TIM-3 [85], downregulation of antigen presentation caused by mutations in genes encoding Janus kinase 1/2 (JAK1/2) [86], and progression of T cells to the terminally exhausted state [87,88]. Additionally, resistance to PD-1/PD-L1 ICIs may also be influenced by molecules that modulate PD-1 signaling to promote immune escape, such as galectin-9 (Gal-9), implicating such proteins as putative targets for cancer immunotherapy [89,90].

### 2.4. Immune-Related Adverse Events Associated with PD-1/PD-L1 ICIs

In addition to the abovementioned limitations, there are grave concerns regarding immune-related adverse events (irAEs) associated with PD-1/PD-L1 ICIs. irAEs are systemic toxicities similar to autoimmune responses driven by an overactivation of the immune system [91]. While IV infusion of anti-PD-1/PD-L1 mAb induces swift onset of therapeutic effects [92] and enables them to bypass absorption and degradation constraints encountered by other means of administration [93], it also allows for off-tumor accumulation in healthy tissues that increases the risk of irAEs [94]. The incidence and severity of irAEs depend on the patient, type of ICI, treatment duration, and whether the ICIs are administered as monotherapy or in combination with other drugs [95]. In particular, patients who are male, over 60 years old, regular smokers, and have high body mass indices and autoimmune diseases are at higher risk of developing PD-1/PD-L1 ICI-induced irAEs [96]. It is worth acknowledging that PD-1/PD-L1 ICI-induced irAEs in children have not been well-characterized relative to adults due to the rarity of pediatric cancers, as well as the poor immunogenicity and lack of PD-L1 expression in many pediatric cancers, making PD-1/PD-L1 ICIs less effective in this patient population. Furthermore, children have developing immune systems, and their responses to ICIs can be unpredictable and more severe compared to adults, hindering their use in children [97,98]. Developing comprehensive pediatric-specific guidelines and increasing the number of clinical studies focused on children may improve the safety and efficacy of ICIs in this vulnerable population.

Clinically, irAEs are graded based on severity according to the Common Terminology Criteria for Adverse Events (CTCAE). Grades I-II are mild and generally do not require systemic steroid treatment; grades III-IV are severe and may require steroid administration and/or ICI withdrawal [99,100,101]. PD-1/PD-L1 ICI-induced irAEs are well-characterized in the literature: most irAEs manifest within six months of treatment [95]; dermatologic irAEs are most common (30–40%) [102] and are accompanied by fatigue, headache, joint pain, pneumonitis, diarrhea, hepatitis, and endocrinopathies [95]; the incidence of irAEs with PD-1/PD-L1 monotherapy is over 60% and even higher when PD-1 ICIs are combined with CTLA-4-targeted ICI [102]; and fatal irAEs occur in 0.36% of patients treated with anti-PD-1, 0.38% with anti-PD-L1, and 1.23% with anti-CTLA-4 combination therapy and are often attributable to pneumonitis (35%), hepatitis (22%), or neurotoxicity (15%) [102]. Unfortunately, in addition to irAEs being potentially fatal, ICI treatment must be discontinued even in patients whose tumors are responding to therapy if the irAEs are deemed severe enough. Thus, developing strategies to minimize the incidence and grade of irAEs is of profound importance for harnessing the full therapeutic potential of PD-1/PD-L1 ICIs.

irAEs caused by PD-1/PD-L1 ICIs are attributed to the recognition of auto-antigens common to healthy and malignant tissues by effector T cells that are disinhibited by ICI therapy [102]. Although it has been reported that PD-1/PD-L1 ICIs exert their effects primarily within the TME, secondary lymphoid tissues, and tumor-draining lymph nodes (TdLNs) [103], the systemic nature of irAEs indicates that re-activated T cells also exert their effector functions elsewhere, evident by off-tumor toxicities in organs throughout the body. The clinically approved mode of ICI delivery largely accounts for this phenomenon since mAb infused into patients does not selectively bind to PD-1/PD-L1 in tumors, and IV administration does not favor the accumulation of mAb in tumor tissues [104]. In addition, mAb has been described to have low penetrance into tumor tissues [105,106], which may necessitate the use of relatively high doses to achieve adequate tumor delivery and desired anti-tumor responses, increasing the risk of irAEs. These ICI delivery-related limitations contribute to systemic overactivation of auto-reactive effector T cells that cannot differentiate malignant from healthy cells [107]. Since irAEs are common and limit the efficacy of PD-1/PD-L1 ICIs in cancer patients, innovative strategies to direct the delivery of its therapeutic effects specifically to tumor tissues are needed to maximize its benefits.

## 3. Alternative Strategies for Tumor-Directed Delivery of PD-1/PD-L1 ICIs in Cancer

To improve the toxicity profile of IV-administered PD-1/PD-L1 ICIs, several targeted drug delivery methods have been developed and studied extensively. Since the mode of administration will greatly influence biodistribution and intratumoral (IT) bioavailability, alternative means of delivering ICIs must take this into consideration. Herein, we review four investigational methods for enhancing tumor-targeted delivery of PD-1/PD-L1 ICIs, namely, locoregional, oncolytic virus-, nanoparticle-, and ultrasound and microbubble-mediated administration techniques (summarized in Table 1). These delivery methods are among the most commonly cited strategies for directing the therapeutic effects of ICIs preferentially to tumor tissues in an attempt to minimize irAEs. In addition to discussing the application of these approaches for delivering PD-1/PD-L1-targeted mAb, we also describe small molecules, peptides [108], siRNA [109], and other agents being developed as potential modes of inhibiting the PD-1/PD-L1 signaling axis for ICI therapy. Considering the breadth of research on alternative methods for delivering PD-1/PD-L1 ICIs, this article is not intended to be a comprehensive review; rather, we aim to provide an update on recent progress made toward establishing new delivery strategies that use different variations of these techniques.

## 4. Locoregional Drug Delivery (LDD)

### 4.1. LDD Concept

**Figure 2:**
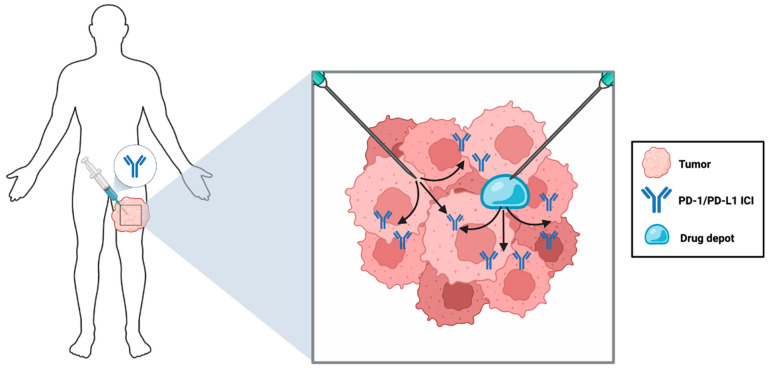
Schematic illustrating LDD for PD-1/PD-L1 ICIs. LDD of PD-1/PD-L1 ICIs involves either direct injection of the ICI into the tumor or the injection of a drug depot intra- or peri-tumorally for sustained release of the ICI.

While many tumor-targeted PD-1/PD-L1 ICI delivery methods aim to localize the drugs using carrier vehicles and tumoritropic agents as discussed in subsequent sections, the simplest strategy is to directly deposit the mAb into the tumor (i.e., IT delivery). This alternative to IV administration is referred to as locoregional drug delivery (LDD). In general, the LDD of ICIs can be grouped into two categories, one involving direct injection of ICIs into the tumor and another involving the placement of slow-release depots intra- or peri-tumorally for extended infusion of ICIs [110]. In principle, both of these strategies should concentrate ICIs inside the tumor to maximize therapeutic efficacy and spare healthy tissues by minimizing off-tumor toxicity (Figure 2).

Conventional LDD administration using percutaneous injection guided by visual inspection is effective but limited in that the tumor must be palpable and accessible. As such, this method is primarily employed for skin cancers such as melanoma [111,112,113]. However, current technologies utilizing ultrasound (US) and computed tomography (CT) have enabled researchers to safely and accurately target almost any tumor mass in the body [114,115,116], providing opportunities to use them for LDD of PD-1/PD-L1 ICIs. US enables real-time imaging of the tumor and the needle while the patient is anesthetized [110], and for malignancies such as lung cancer where US waves are scattered by air in the lungs, or deeper tumors where US imaging is not effective, CT scans can be used to guide the placement of needles for precise tumor targeting [114]. Encouragingly, abscopal effects against distal metastases have been documented upon LDD of immunotherapies such as resiquimod (a toll-like receptor 7/8 (TLR7/8) agonist) and IL-2 in preclinical trials [117], further supporting the idea that LDD could be an effective approach for ICI delivery. Although there is a relative paucity of preclinical trials that have specifically attempted the delivery of PD-1/PD-L1 ICIs by LDD, existing studies, as we describe below, will likely inspire additional trials and evidence to support future clinical translation of this ICI delivery approach in certain cancer contexts.

### 4.2. LDD Variations for Tumor-Targeted ICI Delivery and Evidence of Efficacy

As stated above, the simplest form of LDD is direct IT injection of the therapeutic agent. A study led by Francis et al. compared the efficacy of IT versus intraperitoneally (IP) administered mAb targeting PD-1 and CTLA-4 in a subcutaneous model of B16 melanoma in syngeneic mice. The results indicated that IT administration suppressed tumor growth and prolonged overall survival to a significantly greater extent than ICIs delivered IP. In contrast to IP-mediated ICI delivery, LDD by IT injection was associated with mAb accumulation in the TME and TdLNs, as well as an increase in CD8^+^ tumor-infiltrating lymphocytes (TILs), demonstrating the superiority of LDD drug delivery [118]. LDD of ICIs may be especially beneficial in cancer patients with organ transplants as it could reduce the occurrence of transplant rejection by systemically activated T cells [119]. To model ICI treatment in transplant patients with cancer, Dang et al. investigated the efficacy of an IT-administered anti-PD-1 mAb combined with an anti-TLR9 agonist (Class B CpG oligonucleotide) in mice with cardiac allografts and subcutaneous B16 tumors. The TLR9 agonist was used as an additional anti-tumor agent to activate the innate immune system. Encouragingly, IT administration of the anti-PD-1 mAb with the TLR9 agonist did not result in allograft rejection but stimulated the recruitment of activated CD8^+^ TILs into the TME and induced B16 tumor regression. IP administration of the same drugs resulted in a marginal suppression of tumor growth, but also caused allograft rejection, suggesting that IT administration of ICIs offers enhanced safety in the context of transplantation [120]. In alignment with these findings, a recent phase I clinical trial testing the safety of IT and intracavitary (IC, another form of LDD whereby drugs are directly injected into a resection cavity during surgery) administration of anti-PD-1 (nivolumab) and anti-CTLA-4 (ipilimumab) mAb in 46 glioblastoma patients revealed that delivering these ICIs with either method was feasible and only caused mild irAEs including fatigue, fever, and headaches [121]. Together, these preclinical and clinical studies suggest that using LDD for PD-1 ICIs is not associated with lethal irAEs and has the potential to promote tumor regression in multiple cancer types.

A creative variation of LDD is the use of microneedles instead of hypodermic needles for the delivery of PD-1/PD-L1 ICIs. Microneedle patches are a transdermal drug delivery system consisting of micro-scaled needles attached to a patch that is applied to the skin [122]. Since they provide a painless method for drug delivery, they have high patient adherence and have been used to treat numerous pathologies [123]. Yang et al. engineered a custom hybrid core-shell microneedle system (CSMN) to co-deliver an anti-PD-L1 mAb with an inhibitor of 1-methyl-D, L-tryptophan (1-MT) that activates T cells, to subcutaneous B16 tumors in mice. The anti-PD-L1 mAb was concentrated at the tip of the microneedles via electrostatic interactions, and 1-MT was stably incorporated into the needle core using polyvinyl alcohol (PVA). When tumor-bearing mice were treated using either CSMN or traditional IT needles, the former led to better suppression of tumor growth and longer survival. The authors speculated that this was related to sustained drug release from the skin into the tumors instead of a single bolus infusion. Furthermore, the anti-tumor effects of CSMN were again linked to increased intratumoral CD8^+^ T cell infiltration, explaining the retardation of tumor growth by combined PD-L1 and 1-MT inhibition [124]. This study provides evidence to suggest that anti-tumor efficacy is greater with sustained release than with immediate release of PD-L1 blockade administered by LDD.

Another LDD strategy for sustained release of PD-1/PD-L1 ICIs involves using hydrogels as drug depots for sustained intra- or peri-tumoral ICI release. Hydrogels are a hydrated three-dimensional network of hydrophilic polymers strengthened by physical or chemical crosslinking [125]. Their high water content allows the capture of water-soluble cargo, and the crosslinked polymer network can be easily modified to control the release of virtually any entrapped cargo [126]. Supramolecular hydrogels are physically crosslinked by non-covalent bonds such as hydrogen bonds, electrostatic interactions, π-π interactions, and van der Waals forces. Since these interactions are dynamic and reversible, they are capable of releasing cargo at a slow, controlled rate [126]. In contrast, chemically crosslinked hydrogels are composed of covalently linked molecules that assemble into a network of rigid polymers, from which cargo is liberated by slow diffusion [127,128]. Shi et al. evaluated the anti-tumor efficacy of an IT-implanted supramolecular polypeptide hydrogel co-loaded with an anti-PD-L1 mAb and doxorubicin (Dox) in mice with B16 tumors. This treatment modality resulted in tumor growth inhibition and conferred better survival benefits than IT-administered PBS, free anti-PD-L1, anti-PD-L1-loaded hydrogel, free Dox, Dox-loaded hydrogel, and combined free anti-PD-L1 plus Dox controls. In addition, incorporating the therapeutics into a hydrogel extended the duration of the anti-tumor effects relative to IT-administered free drug controls. Importantly, mice treated with LDD in the form of drug-loaded hydrogels exhibited reduced systemic toxicity as measured by weight loss compared to those treated with IT-administered free anti-PD-L1 and Dox. Thus, these results suggest that this mode of LDD can sustain effective drug release, prolong tumor responses, and also reduce irAEs [129]. However, a more thorough toxicological analysis including dermatological and hematological examinations to evaluate clinically relevant irAEs would strengthen this idea. Likewise, Liu et al. formulated supramolecular hydrogels encapsulating Dox and a peptide that binds to and antagonizes PD-L1 (D-PPA-1) and injected them IT into subcutaneous CT26 colon tumors in mice. This study also demonstrated that LDD using hydrogels effectively induced tumor regression and reported that hydrogel-incorporated therapeutics were longer-lasting than IT-injected free drug controls. Specifically, they robustly detected D-PPA-1 and Dox 24 h post-injection in the hydrogel-incorporated group, but could not reliably detect free D-PPA-1 and Dox-administered IT. Furthermore, they observed enhanced tumor infiltration of CD8^+^ T cells, and TNF-α and IFN-γ secretion in mice treated with hydrogel-incorporated D-PPA-1 and Dox compared to mice treated with hydrogels loaded with either D-PPA-1 or Dox alone, owing to the combinatory therapeutic effects of Dox and PD-L1 blockade [130].

Intriguingly, ICI-loaded hydrogels that assemble in situ (i.e., within the injected tissue) upon IT or peri-tumoral injection have yielded similar findings. For instance, Wang et al. tested the anti-tumor efficacy of in situ-forming supramolecular hydrogels loaded with an anti-PD-1 mAb and camptothecin (topoisomerase I inhibitor) in a subcutaneous model of murine GL-261 brain cancer and an orthotopic model of 4T1 breast cancer. They demonstrated that IT injection of these hydrogels increased CD8^+^ TIL counts, induced tumor regression, prolonged retention of the anti-PD-1 mAb (detectable at 15 days post-injection), and reduced toxicity as no weight loss was observed [131]. In a different study, peri-tumoral co-delivery of anti-PD-1 + CTLA-4 mAb entrapped by an in situ crosslinked thermosensitive hydrogel that gradually released the mAb at physiological temperatures, significantly stunted tumor growth compared to peri-tumoral co-delivery of free anti-PD-1 + CTLA-4 as reported by Kim et al. Moreover, while the hydrogel anti-PD-1 + CTLA-4 group did not exhibit any signs of liver toxicity as measured by serum alanine aminotransferase (ALT) and aspartate aminotransferase (AST) levels, the free drug group had elevated ALT/AST levels indicative of toxicity. This suggests that the incorporation of ICIs into hydrogels improves their safety [132]. Altogether, these LDD studies illustrate that (1) IT LDD does not cause systemic toxicities that are observed upon IV administration of PD-1/PD-L1 ICIs, (2) IT administration effectively induces tumor regression and prolongs survival in several subcutaneous tumor models, and (3) the use of hydrogels for LDD greatly enhances the efficacy of IT administered PD-1/PD-L1 ICIs compared to IT free drug infusions. However, it is important to note that none of these studies compared the efficacy and tolerability of IT-administered PD-1/PD-L1 ICIs to conventional IV administration, which is needed to assess whether IT administration achieves similar anti-tumor efficacy with a lower incidence of irAEs.

### 4.3. Challenges with LDD of PD-1/PD-L1 ICIs

Although LDD may concentrate ICIs within tumor tissues, there are concerns regarding the distribution of ICIs within the tumor since a single bolus injection may not be evenly distributed across the entire tumor mass. As such, this could limit the therapeutic potential of ICI delivered with LDD. To circumvent this limitation, the utilization of a multi-needle injection system may be necessary to achieve a more diffuse distribution of ICIs within tumors [133]. Moreover, manual injection of ICIs via LDD is prone to human error including leakage of the ICI into surrounding tissues or blood vessels, increasing the risk of irAEs. Biological variations such as differences in the stiffness of the tumor stroma between different cancers and patients with the same cancer could also render IT injections unreliable [134]. The development of automated injection instruments and/or the use of sustained-release platforms (e.g., hydrogels) may help to mitigate this issue.

Perhaps the most important limitation of using LDD for ICI delivery is that its utility may be largely restricted to easily accessible, subcutaneous, or cutaneous tumors such as melanoma. The relative paucity of preclinical studies demonstrating the efficacy of LDD in tumors that are not superficial provides evidence of this limitation. As such, image-guided injection using magnetic resonance imaging (MRI), CT scans, and US should be explored more extensively to broaden the applicability of this effective technique to a larger fraction of cancer patients.

## 5. Oncolytic Virus (OV)-Mediated Delivery

### 5.1. OV Concept

**Figure 3:**
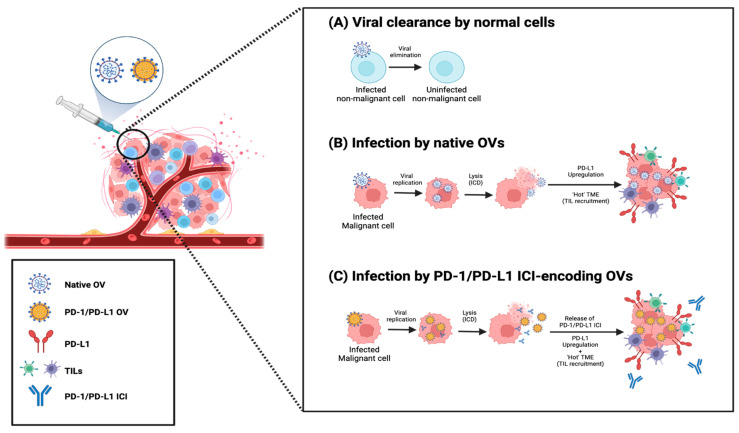
Diagram illustrating OV-mediated delivery of PD-1/PD-L1 ICIs. While non-malignant cells are capable of clearing OVs upon infection because they have a functional antiviral response (**A**), many cancer cells are susceptible to OV infection and replication due to dysfunctional antiviral responses, which can be potentiated by genetic modifications to OVs that promote their replication specifically in tumor cells. OV-induced oncolysis causes immunogenic cell death (ICD) of tumor cells which releases viral progeny and primes the TME for PD-1/PD-L1 ICIs by recruiting anti-tumor lymphocytes and upregulating PD-L1 on tumor cells (**B**,**C**). OVs can also be genetically engineered to encode anti-PD-1/PD-L1 agents such as antibodies, directing their synthesis, release, and therapeutic effects preferentially to tumor tissues (**C**).

Oncolytic viruses (OVs) have emerged as an exciting method for delivering ICIs and other immunotherapies, spurred by the FDA’s approval of a herpes simplex virus (HSV)-derived OV called talimogene laherparepvec (T-VEC) for the treatment of melanoma [135]. Simply put, OVs are native or genetically modified viruses that preferentially replicate within and lyse cancer cells (Figure 3). Currently, two classes of OVs exist. Class I OVs, including parvovirus, myxoma virus (MYXV), reovirus, Newcastle disease virus (NDV), and Seneca Valley virus (SVV), are unmodified viruses. The selective replication of these viruses in cancer cells is attributable to the fact that tumors often have defective antiviral responses while non-malignant cells rapidly respond to eliminate OVs [136]. Class II OVs are genetically modified viruses such as recombinant poliovirus (PV), measles virus (MV), vaccinia virus (VACV), adenovirus (AV), HSV, and vesicular stomatitis virus (VSV) [137]. These OVs can be engineered to enhance their safety profiles by including genetic modifications that reduce viral pathogenicity by limiting their replication in normal cells [138,139]. In addition, synthetic OVs can be employed as vectors for delivering therapeutic cargo by engineering them to encode immunogenic molecules that potentiate anti-tumor responses [137,140].

While the precise mechanisms governing their oncolytic properties are strain- and modification-dependent, by lysing cancer cells, any OV strain should be capable of inducing immunogenic cell death (ICD) which is highly inflammatory: lysis of infected cancer cells causes liberation of tumor-associated antigens (TAAs), cellular damage-associated molecular patterns (DAMPs), and pathogen-associated molecular patterns (PAMPs), which recruit and activate lymphocytes, subsequently enhancing anti-tumor immunity [141]. Given the nature of viruses, OVs also release viral progeny upon lysing host cancer cells, leading to infection of neighboring cancer cells, thereby maintaining and propagating the anti-tumor response in the TME [142].

The potential benefits of OV-mediated ICI delivery include tumor-specific delivery, which might lead to higher local concentrations of ICIs in the TME and reduced systemic exposure, potentially decreasing the incidence and severity of irAEs. Since it has been challenging to deliver sufficient quantities of OVs to tumors with IV administration, direct IT inoculation has been the only effective route of delivery in patients and most preclinical models to date [143]. As such, it can be considered a form of locoregional therapy. In principle, leveraging OVs to deliver PD-1/PD-L1 ICIs preferentially to tumor tissues is a rational approach since the immunogenic nature of OVs should also prime the TME to potentiate ICI-induced anti-tumor responses. This addresses one of the limitations of PD-1/PD-L1 ICIs—that their effects are dependent on the tumor being immunologically ‘hot,’ containing an adequate number of TILs such as tumor-reactive T cells that can be de-repressed in the TME. Indeed, immunologically ‘cold’ tumors show limited response to ICIs [144,145]. As explained above, even without genetic modifications, the mere process of OV infection and lysis of cancer cells induces ICD that can create a ‘hot’ TME with increased infiltration of leukocytes [137]. Moreover, OV infection in a subpopulation of cancer cells within a tumor often results in upregulation of PD-L1 in neighboring cancer cells via an IFN-dependent mechanism [146,147], further rationalizing the use of OVs to deliver PD-1/PD-L1 ICIs. Collectively, these properties unique to OVs distinguish them from basic locoregional injections of PD-1/PD-L1 ICIs.

### 5.2. OV Variations for Tumor-Targeted ICI Delivery and Evidence of Efficacy

Multiple studies have utilized genetically modified OVs encoding PD-1/PD-L1-targeted antibodies or antibody fragments to focus ICI delivery to tumors and enhance their efficacy in vivo. Vitale and colleagues constructed an oncolytic AV carrying an anti-PD-L1 antibody expression cassette (Ad5Δ24-anti-PD-L1-scFv) and evaluated its anti-tumor effects in subcutaneous B16.OVA tumors. As expected, IT inoculation of Ad5Δ24-anti-PD-L1-scFv resulted in superior tumor growth inhibition and prolonged survival compared to the unmodified parental Ad5Δ24 strain. This therapeutic effect was associated with an enrichment of infiltrating CD8^+^ T cells attributed to OV- and anti-PD-L1-mediated anti-viral and anti-tumor responses [148]. Comparable findings have been reported by other groups using different strains of OVs to deliver anti-PD-1 or anti-PD-L1 antibodies [149,150,151,152]. For example, Veinalde et al. engineered an oncolytic MV encoding either an anti-PD-1 or anti-PD-L1 mAb. They found that this MV endowed long-lasting immunological memory against subcutaneous MC38cea colon tumors. In their study, mice in which IT MV-mediated delivery of ICI induced complete remission of these tumors also rejected secondary tumors upon rechallenge with MC38cea injected into the flank contralateral to the primary tumor site. The induction of immunological memory was also evident by an increase in effector memory T cells within the primary tumors [153].

OV-encoding ICIs have also been explored in combination therapies. Xie et al. [154] reported on their combination of an HSV-1 OV encoding an anti-PD-1 mAb (VT1093M) with a distinct HSV-1 OV expressing IL-12 (VT1092M), a cytokine involved in T and NK cell activation [155], in CT26 colon tumors. They found that IT delivery of these OV as monotherapies resulted in transient tumor regressions followed by gradual tumor progression in most mice; but, when VT1093M and VT1092M were co-injected IT, durable tumor regressions were observed in all mice, accompanied by the largest increase in tumor-infiltrating CD4^+^ and CD8^+^ T cells and overall survival rates. They also demonstrated that this anti-tumor effect was cancer-specific: when mice that experienced CT26 tumor regression upon VT1093M + VT1092M treatment were rechallenged with CT26 cells or H22 hepatocellular carcinoma cells, only H22 formed tumors [154]. These findings provide proof of concept that ICI-encoding OVs can be combined with other OVs delivering distinct immunomodulatory therapeutics to enhance anti-tumor efficacy and long-lasting memory.

Another OV ICI combination therapy approach involves encoding multiple therapeutic components within a singular OV. Chouljenko et al. generated an oncolytic HSV-1 strain encoding IL-12 and -15, the alpha subunit of the IL-15 receptor, as well as an anti-PD-L1 blocking peptide, and found that it elicited robust anti-tumor effects when administered IT to treat subcutaneous CT26 and A20 B cell lymphoma tumors in syngeneic mice. Differentiating this study from others that have tested OV-mediated delivery of ICIs, was the utilization of cynomolgus monkeys (*Macaca fascicularis*) to evaluate toxicities, which were not considered in the OV studies discussed above. Chouljenko et al. intramuscularly treated four monkeys per group with either PBS or the OV at doses of 2.97 × 10^7^, 2.97 × 10^8^, and 2.97 × 10^9^ PFU/animal. They observed no signs of toxicity associated with OV treatment based on measurements of body weight, temperature, electrocardiograms, cytokines, and T cells. They also performed a necropsy examination 15 days post-treatment and found no evidence of toxicity based on macroscopic examination [156]. While these findings are encouraging, comparing toxicities in OV-ICI treated animals to those treated with conventional IV administration of ICI is imperative for understanding whether utilization of OVs as immunogenic vectors for ICI delivery elicits similar or superior therapeutic efficacy with reduced incidence of irAEs. Nevertheless, these recent advances in OV-mediated ICI delivery have substantially expanded the options available for delivering PD-1/PD-L1 ICIs preferentially to tumor tissues, whilst simultaneously sensitizing them to PD-1/PD-L1 ICIs by stimulating ICD. Future studies should not only focus on toxicological evaluations but also on investigating whether PD-1/PD-L1 ICIs could be co-delivered with other ICIs such as those targeting CTLA-4. This is because targeting multiple inhibitory immune checkpoints concomitantly often induces stronger anti-tumor effects, which is why PD-1 and CTLA-4 ICI combinations are standard of care in patients with metastatic melanoma, advanced/recurrent NSCLC, malignant pleural mesothelioma, amongst others [157]. However, ICI combination therapy may also increase the risk of irAEs [158], reinforcing the need for extensive toxicological analyses.

It is also worth mentioning that several recent studies have investigated co-administration of PD-1/PD-L1 ICIs and native OVs [147,159,160,161]. Theoretically, OV-induced ICD should enhance the anti-tumor efficacy of ICI by increasing binding sites for ICIs (i.e., upregulating PD-L1 expression) specifically within the TME where OVs exert their lytic effects, and by prolonging ICI residence time. Although this method does not direct the delivery of ICI mAb specifically to tumor cells, it may indirectly focus their effects preferentially on tumor tissues and reduce ICI-associated toxicities via tumor-specific lysis and ICD. For instance, treatment of MC38-Luciferase colon tumors with an oncolytic vaccinia virus (OVV) co-administered with an anti-PD-L1 mAb effectively inhibited tumor growth and prolonged survival of mice compared to those treated with OVV or anti-PD-L1 mAb alone [159]. Complementing this preclinical trial, a phase 1b clinical trial revealed that combination therapy with IT T-VEC and an approved anti-PD-1 mAb (pembrolizumab) was effective in advanced melanoma patients: 82% of injected patients experienced >50% reductions in tumor volume without any dose-limiting toxicities, and this was accompanied by an increase in tumoral PD-L1 expression, CD4^+^ and CD8^+^ T cells, T cell PD-1 expression, and memory T cell density [147]. Lastly, a preclinical trial also explored the efficacy of OV combination therapy using a murine T-VEC strain (OncoVEC^mGMCSF^) with an IP-administered anti-PD-L1 mAb and radiotherapy (RT) against subcutaneous B16-F10-nectin tumors. The OV + RT treatment decreased tumor burden to a greater degree than either monotherapy and recruited CD8^+^ T cells, providing evidence that OV + RT transformed the TME into an immunologically ‘hot’ state. The compensatory increase in PD-1/PD-L1 signaling was abrogated by the anti-PD-L1 mAb, yielding an effective triple combination therapy that further inhibited tumor growth [161]. Based on strong preclinical evidence of their efficacy and tolerability, an abundance of clinical trials are currently testing co-administration of OVs and PD-1/PD-L1 ICIs in cancer patients (ClinicalTrials.gov trials: NCT06196671, NCT05122572, NCT05222932, NCT05346484, and NCT02798406). Collectively, these studies provide evidence that regimens using OV to prime the TME for combination with PD-1/PD-L1 ICI, enhance the therapeutic effects of PD-1/PD-L1 blockade.

### 5.3. Challenges with OV-Mediated ICI Delivery

Like other tumor-targeted approaches, OVs are also limited by treatment challenges. A major limitation of using OVs to deliver ICIs is their poor bioavailability in tumor tissues due to rapid clearance by the hosts’ reticuloendothelial (RE) system [143]. Since non-replicating viral vectors (e.g., replication-defective AVs) are less immunogenic than replicative OVs, these could be used as alternative OV strains, albeit at the cost of reduced therapeutic potency [162,163]. Clearance of OVs before they reach the tumor significantly limits the mode of OV delivery to LDD by IT injection, and as a result, significantly limits their therapeutic utility. This is reflected by the fact that most preclinical trials have used subcutaneous tumor models to evaluate OV efficacy, suggesting that OV-mediated ICI delivery may only be clinically tractable for superficial cancers such as melanoma. As such, future studies should investigate and report the efficacy of OV ICI therapy in other orthotopic tumor models, including metastatic tumors, to better understand their therapeutic potential. Another obstacle associated with OV-mediated ICI delivery is the possibility of resistance to certain viral strains used to generate OV. For instance, tumor cells may be intrinsically capable of preventing viral entry or inhibiting viral transcription due to epigenetic alterations and upregulation of antiviral proteins [164]. Alternatively, some individuals may carry neutralizing antibodies to specific viruses due to prior infections, repeated exposure to the OV for treatment, or vaccinations, which may lead to OV clearance and reduce the OV’s ability to effectively infect and lyse tumor cells, especially when administered IV [165,166]. Since preexisting viral immunity may diminish treatment efficacy, clinicians may need to personalize OV therapy, for example, by screening patients for biomarkers of immunity to OV strains (e.g., neutralizing antibodies) [165]. However, this could be prohibitive due to the time required to perform such tests, as disease progression could occur before an appropriate OV is identified. Lastly, the use of viruses may incite generalized fear by the public, resulting in poor patient enthusiasm. Extensive evaluation of toxicities associated with using OVs to deliver ICIs and a thorough explanation of the risks and benefits could ease patient distress.

## 6. Nanoparticle (NP)-Mediated Delivery

### 6.1. NP Concept

Given that IV administration of ICIs is simple, relatively non-invasive, and effective, significant efforts have been made to engineer delivery vehicles to target ICIs more specifically to cancer cells in order to avoid systemic drug deposition and associated irAEs. The idea of shielding ICIs in circulation while liberating them preferentially at tumor sites has gained traction. To this end, researchers have developed NPs to encapsulate anti-cancer drugs to enhance tumor-specific delivery [167]. NPs are typically defined as particles ranging in size between 1–100 nm that display distinct properties from their macroscale counterparts with the same chemical makeup [168], and many clinically approved NPs are ≥ 50–100 nm in size [169]. Drug-carrier NPs can be classified into three different categories: inorganic, organic, or hybrid NPs. Inorganic NPs include silver, gold, silica, magnetic, and carbon nanotube NPs and are composed of inorganic substances. They are readily amenable to surface modifications, have large surface area-to-volume ratios [170], and have unique optical and magnetic properties [171]. On the other hand, organic NPs are constructed from aggregated organic molecules and include lipid-based and protein-based NPs, as well as synthetic polymers such as poly(lactic- co-glycolic) acid (PLGA) and polyanhydrides [172]. In contrast to inorganic NPs, organic NPs can be engineered to be biocompatible and biodegradable, making them more environmentally friendly and safe [173]. Lastly, hybrid NPs such as metal–organic, metal-phenolic, metallofullerene, and polymer-lipid NPs [174] are conjugates of organic and/or inorganic NPs, and therefore have functional benefits associated with both. Advances in materials science have enabled the synthesis of hybrid NPs with physical modifications that further enhance their biological behaviors by, for example, enabling longer circulation times and higher target specificity [175]. Notably, all three NP categories are being investigated for their utility as carriers of ICIs.

**Figure 4:**
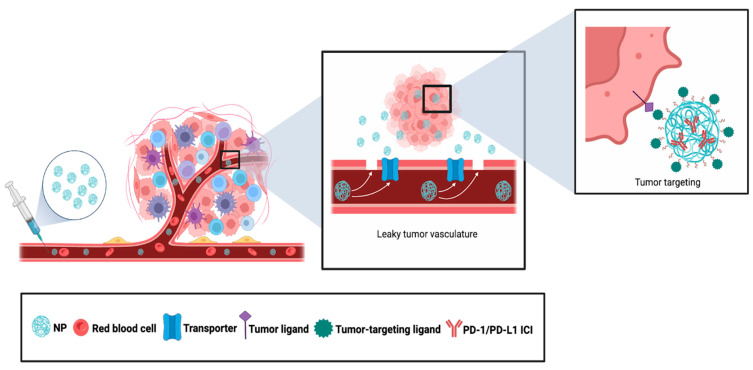
Schematic indicating NP-mediated delivery of PD-1/PD-L1 ICIs. NP-mediated delivery of PD-1/PD-L1 ICIs generally involves IV administration of NPs with the ICI agent encapsulated within the NP or incorporated on the NP surface. After they travel in circulation and make their way to the tumor vasculature, they either passively extravasate into the interstitial space due to inherent leakiness of the tumor vasculature, or they actively traverse the endothelium by transcytosis which can be aided by surface modifications on NPs. Once ICI-loaded NPs have extravasated from the bloodstream, they either passively diffuse through the tumor stroma until they reach the tumor to block PD-1/PD-L1 signaling, or they actively degrade the ECM using functional molecules conjugated on their surface to facilitate migration through the stroma. Notably, tumor-targeting ligands on NPs can further direct them to tumors to enhance their uptake.

The majority of anti-cancer NPs are administered IV [176]. This requires NPs to (1) extravasate from the bloodstream into the interstitial space, (2) migrate through the tumor stroma to access tumor tissues, and (3) interact with the tumor for binding and/or uptake. Currently, two non-mutually exclusive models exist for the extravasation of NPs across the endothelium, both of which contribute to NP uptake and retention preferentially in tumor tissue. The first model called the Enhanced Permeability and Retention effect (EPR effect) proposes that NPs passively diffuse across the endothelial layer due to the abnormal, leaky, and disorganized tumor vasculature and are retained in tumor tissues, at least in part, by a dysfunctional lymphatic system [177,178,179,180]. In essence, the tumor becomes a sink for NPs due to the EPR effect, and this is how NPs are concentrated in tumors. The second model named the Active Transport and Retention effect (ATR effect) postulates that NPs are actively transported across the endothelium by mechanisms including transcytosis. The NPs are then retained preferentially in tumor tissues due to interactions with cellular and acellular factors within the tumor [181]. Since the EPR effect is an inefficient process that requires long circulation times for the NPs to concentrate inside the tumor [182], NP surfaces are often decorated with carrier ligands such as transferrin and albumin which interact with their respective receptors that are overexpressed in tumor endothelial cells to facilitate active extravasation [183]. Penetration across the tumor stroma can be achieved by NP formulations that, for instance, increase interaction with neutrophils migrating to tumors or induce degradation of the ECM [184,185]. Once NPs reach the vicinity of a tumor, they can actively interact with tumor cells through multiple mechanisms, which is usually mediated by incorporating tumor-targeting ligands onto the surface of NPs that bind to proteins specifically overexpressed in tumors (Figure 4) [186]. In addition, stimuli-responsive NPs can be engineered to release their therapeutic cargo upon encountering environmental features characteristic of the TME, such as high acidity, hypoxia, and excessive reactive oxygen species (ROS). In this way, these common properties of tumor tissues can be exploited to liberate drugs from NPs specifically in the TME [187]. There is an ever-expanding literature describing how NPs are being synthesized to overcome biological barriers that impede their delivery to tumor tissues. Although we describe specific examples below, we refer readers to several excellent reviews to gain a thorough understanding of different strategies used for tumor-targeted NP delivery [188,189,190].

Following the FDA approval of Doxil in 1995, a liposome NP loaded with Dox [191], a variety of NPs have been approved for chemotherapeutic delivery including but not limited to DaunoXome (daunorubicin) and Marqibo (vincristine) [192]. Importantly, these NPs have been shown to enhance the uptake of chemotherapeutics preferentially in tumor tissues [193,194,195]. Inspired by this success, establishing effective NP solutions for tumor-targeted delivery of ICIs has become a growing field of research [196,197,198,199]. Numerous studies have demonstrated successful NP-mediated delivery of ICIs to diverse tumor types, as well as associated anti-cancer effects [183,200,201,202,203,204,205,206,207,208]. Here, we describe some of the most recent and compelling examples of effective, NP-mediated strategies for tumor-targeted delivery of PD-1/PD-L1 ICIs.

### 6.2. NP Variations for Tumor-Targeted ICI Delivery and Evidence of Efficacy

Some recent studies investigating NPs as conduits for ICI delivery simply incorporated or encapsulated PD-1/PD-L1 ICIs into NPs. Lim et al. engineered a pH-responsive polymeric NP incorporating an anti-PD-L1 mAb (anti-PD-L1-NP) to block PD-1/PD-L1 signaling in a subcutaneous MC38 colon cancer model. The acidic nature of the TME relative to non-malignant tissue, which has been observed in many tumors [209], was leveraged to release the ICI in tumor tissues. The authors observed that anti-PD-L1-NPs successfully delivered anti-PD-L1 mAb to tumors in mice following IP administration. Furthermore, compared to IP-administered saline and free anti-PD-L1 mAb controls, anti-PD-L1-NP induced superior tumor regressions, and immunohistochemistry (IHC) on treated tumors revealed an increase in tumor-infiltrating CD8^+^ and CD4^+^ T cells only in the anti-PD-L1-NP group. Importantly, there were no discernable signs of toxicity upon hematoxylin and eosin (H&E) staining of major organs and measurement of body weight, indicating the safety of anti-PD-L1-NPs [210]. Similar ICI incorporation strategies have been used with small hairpin RNA (shRNA) [211] or small interfering RNA (siRNA) [212] targeting PD-1/PD-L1, and PD-L1 binding peptides [213]. Since RNA therapeutics such as shRNAs and siRNAs are not stable in circulation, NP encapsulation offers an effective delivery solution by protecting them from degradation and premature release [214,215]. Unsurprisingly, these studies consistently demonstrated tumor delivery of NPs, blockade of PD-1/PD-L1 signaling, and associated therapeutic benefits including tumor growth inhibition and prolonged survival times of NP-ICI-treated mice compared to controls.

In addition to using NPs to deliver ICI monotherapy, they can also be used to deliver ICI combination therapy via simultaneous loading with PD-1/PD-L1 ICIs and other therapeutic agents. Liu et al. engineered NPs loaded with the platinum-based chemotherapy oxaliplatin (OXA) and an anti-PD-L1 peptide (CLPoo2), which was conjugated to NPs by a peptide linker (denoted CMFn@OXA). These NPs were derived from ferritin, a protein that naturally forms a cage-like structure suitable for surface functionalization/drug loading and binds transferrin receptor 1 (TfR1) overexpressed on tumor cells, to facilitate tumor-targeted delivery. CMFn@OXA NPs achieved the tumor-targeted release of CLPoo2 and OXA in subcutaneous MC38 tumors in the following manner: upon reaching the TME by the EPR effect, CLPoo2 was liberated through cleavage of the peptide linker by matrix metalloproteinases abundant in the TME, allowing it to block PD-1/PD-L1 interactions within the tumor; OXA was released upon NP internalization, presumably through TfR1-mediated endocytosis, to elicit tumor killing. IV administration of CMFn@OXA significantly suppressed tumor growth, decreased tumor weight, and prolonged survival relative to control tumors treated with IV administration of PBS, free OXA, HFn@OXA NPs (which did not carry CLPoo2), and OXA with IP injection of αPD-L1 mAb. These therapeutic effects were associated with increased CD4^+^ and CD8^+^ T cells, and IFN-γ and TNF-α in tumor tissues, indicating that CMFn@OXA NPs induced a strong anti-tumor immune response. H&E stains of major organs including the liver and lungs, and serum biochemical analyses revealed no toxicities, indicating that these NPs were safe and well-tolerated [216]. A similar study by Mu et al. engineered NPs co-loaded with Dox and siRNA targeting PD-L1 to deliver chemoimmunotherapy. The authors observed an enhanced accumulation of NPs in tumor tissues which was associated with the suppression of tumor growth in a prostate cancer model of bone marrow metastasis [217]. Using a different combinatorial approach, Zhang et al. engineered two different types of NPs: one loaded with an anti-PD-L1 small molecule inhibitor (BMS-1 NP), and another loaded with docetaxel (DTX) chemotherapy and VTX-2337, a toll-like receptor 8 agonist capable of polarizing M2 macrophages into the M1 anti-tumor phenotype (DTX@VTX NP). Co-administration of these two NPs activated both arms of the immune system in subcutaneous 4T1 breast tumor-bearing mice. The innate immune response was activated by DTX@VTX NP evident by a decrease in MDSCs and polarization of M2 macrophages to an M1 phenotype in the TME. The adaptive immune response was activated by BMS-1 NP demonstrated by an increase in tumor-infiltrating CD8^+^ T cells and upregulation of IFN-γ. Simultaneous IT delivery of these two NPs led to a potent anti-tumor response: it elicited a 90.5% tumor inhibition rate, compared to 69.8% for BMS-1 NPs and 48.2% for DTX@VTX NPs alone [218].

Interestingly, Liu et al. showed that oral administration of PD-1/PD-L1 ICIs is feasible with NPs, providing a novel and less invasive route of ICI administration compared to IV. They constructed gold NPs incorporating a protein inhibitor of PD-L1 (P-peptide) and encapsulated these NPs with a milk-derived exosomal membrane. This exosome-wrapping approach was utilized to reduce the immunogenicity of the NPs and prevent immune-mediated clearance [219]. These NPs were administered orally to mice bearing subcutaneous MC38 tumors and were shown to accumulate in tumor tissues based on mass spectrometry analyses that detected the gold component of the NPs. Treatment of subcutaneous MC38 tumors with these NPs inhibited tumor progression, which was associated with increased infiltration of CD8^+^ T cells and expression of CD8^+^ T cell-associated inflammatory factors (perforin, GzmA, and GzmB). Importantly, the authors did not detect any systemic toxicities based on weight, blood, and histological examinations of major organs, indicating their tolerability [219]. Collectively, these studies, and many more, have showcased that NPs are a versatile platform for the effective delivery of various forms of PD-1/PD-L1 ICIs to tumors with minimal toxic side effects.

### 6.3. Challenges with NP-Mediated ICI Delivery

Despite their demonstrated abilities to elicit anti-tumor effects, the efficacy of NPs carrying PD-1/PD-L1 ICIs can be limited by physiological obstacles that impair delivery to tumor tissues. For example, EPR-mediated passive uptake of NPs into tumor tissue can be highly variable due to heterogeneity in the tumor-stromal architecture of individual tumors [208], which may diminish access of NPs to tumor tissues, and consequently, their therapeutic effects in some individuals. The ‘protein corona’ that forms and encapsulates NPs in circulation is another physical challenge associated with NP-mediated drug delivery. IV-administered NPs interact with plasma proteins in the blood that form a shell known as the corona. The corona substantially alters the surface properties of NPs, and this can interfere with tumor delivery, especially if there are tumor-targeting ligands conjugated to the surface of the NPs. While the incorporation of PEG into NPs (PEGylation) has been proposed to restrict corona formation via steric hindrance, it is not perfect [208,220,221], making it difficult to predict the fate or localization of IV-administered NPs. Additionally, NPs larger than 5–10 nm in diameter are prone to clearance by the RE system in the liver and spleen [170], which lowers the effective dose reaching tumor sites. Unfortunately, using NPs less than 10 nm in size is not a simple solution since synthesizing polymeric and lipid-based NPs within this size range is technically challenging, and smaller NPs have reduced drug loading capacity, which reduces the therapeutic payload delivered to the tumor. Strategies that minimize recognition and/or clearance of NPs by the RE system may be required to resolve this issue. To this end, some studies have shown that coating NPs with anti-phagocytic membrane proteins such as CD47 can inhibit phagocyte-mediated clearance of NPs and prolong their time in circulation and retention in tissues [222]. Other factors have also been described to negatively affect the efficacy of NPs, including their penetration through the extracellular matrix (ECM) and their internalization by targeted cancer cells. However, as highlighted above, NP formulations with more favorable physical properties such as size, shell composition, and charge are being developed to overcome these delivery barriers [223,224,225]. Although the studies summarized above did not report any dose-limiting toxicities, there are also concerns regarding potential toxicities caused by NPs. Mechanistically, NPs can cause toxicity by, for example, activating an inflammatory response or inducing the production of ROS that are damaging to tissues [226]. Thus, studies to robustly demonstrate the safety of NP-mediated ICI delivery approaches will be required for their translation into the clinic.

## 7. Ultrasound and Microbubble (USMB)-Mediated Delivery

### 7.1. USMB Concept

The inefficiency of EPR-mediated uptake of drugs into tumor tissues, combined with the extremely dense tumor stroma in many solid tumors such as pancreatic cancer [227], create significant physical barriers that limit the ability of ICIs to penetrate into tumor tissues regardless of the mode of delivery (i.e., NP or LDD) [228,229]. A promising strategy that can expedite and increase extravasation and migration of therapeutic agents through the dense tumor stroma and into tumor tissues while reducing systemic toxicity involves the use of ultrasound and microbubbles (USMB). MBs such as Optison^TM^ and Definity^TM^ are microspheres typically within the range of 1–10 μm in diameter, composed of a biologically inert gas core (e.g., perfluorocarbons) enclosed by a protein, lipid, or polymer shell for structural stability [107]. In the clinic, their ability to undergo mechanical oscillations known as cavitation, where they expand and contract rapidly in response to a US field, enhances diagnostic imaging by increasing contrast between the blood and surrounding tissue [230]. However, as a therapeutic modality, MBs have the capacity to be conjugated to, loaded with, or co-administered with therapeutic agents, allowing them to be repurposed as drug carriers [107,231]. Like NPs, their amenability to carrying specific cargoes can be modulated by altering the chemical composition of the MBs. For instance, incorporation of cationic lipids into the MB shell allows them to bind anionic cargo such as DNA and RNA to deliver genetic therapies [232]. Furthermore, the biological effects of US-induced MB cavitation can be leveraged for USMB-mediated drug delivery as we describe below.

**Figure 5:**
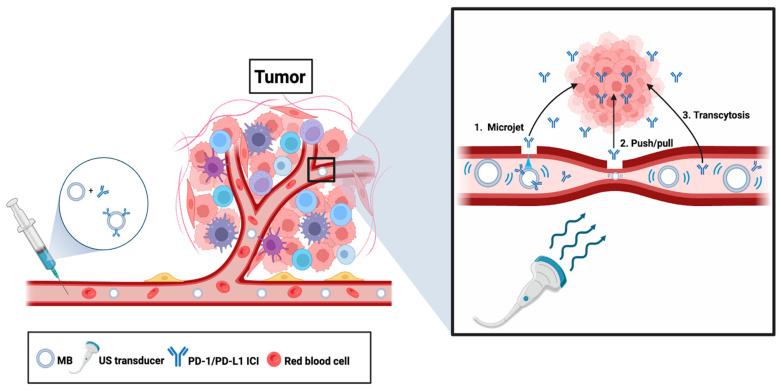
Illustration of USMB-mediated delivery of PD-1/PD-L1 ICIs. USMB-mediated delivery of PD-1/PD-L1 ICIs involves IV infusion of MBs conjugated to, loaded with (incorporated into the shell), or co-administered with the ICI. The MBs travel throughout the body, some of which perfuse the tumor. When MBs within tumor tissues are exposed to a US beam, they cavitate, promoting extravasation and migration of the ICIs by opening transient pores in membranes, pushing and pulling on the endothelium, and activating endocytic pathways in cancer cells. As such, applying US to tumor tissues can facilitate tumor-directed uptake of USMB-delivered ICIs.

Drug delivery using USMB involves IV administration of drugs with MBs, followed by the application of US to cavitate the MBs in targeted tissues. Cavitation of MBs is dictated by their physical properties including size, shell composition, type of gas core, and surface modifications because they alter how MBs respond to US waves [232]. For instance, phospholipid-based MBs are most commonly used for drug delivery as their thin and soft shells offer superior acoustic responses relative to protein or polymer-based MBs which have thicker and stiffer shells [107,233]. Importantly, at low acoustic pressures, MBs oscillate in a stable manner (stable cavitation) which causes fluid microstreaming; at high pressures, they oscillate violently and implode (inertial cavitation), producing shock waves and high-velocity microjets [234]. These mechanical forces exert shear stress on cells, contributing to an increase in paracellular and transcellular drug permeability by rearranging tight junctions [235], activating endocytic pathways [236], and sonoporation or the opening of transient pores on cell membranes [231,237]. Although the exact mechanism is still unclear, MB cavitations also cause vessel distention and invagination, which is hypothesized to facilitate the movement of cargo through the ECM [238] (Figure 5). Remarkably, these transport effects are restricted to tissues where the US is applied, which enables spatiotemporal control of drug release and uptake, minimizing systemic toxicity. As such, drugs can be targeted to tumor tissues by directing the US to the tumor site. Some studies have utilized USMB to enhance the delivery of NP-immunotherapy or NP-chemotherapy complexes [239,240], highlighting its ability to facilitate extravasation of molecular cargo in circulation. Others have demonstrated the practicality of this approach for delivering certain chemotherapies [241], and as we discuss below, USMB is also being explored as an alternative mode of delivering PD-1/PD-L1 ICIs in cancer patients.

### 7.2. USMB Variations for Tumor-Targeted ICI Delivery and Evidence of Efficacy

USMB using MBs directly conjugated to ICIs has proven to be an effective method of delivering PD-1/PD-L1 ICIs in multiple tumor models. For example, Kim et al. treated subcutaneous CT26 tumors with IV-infused anti-PD-L1 mAb-conjugated phospholipid MBs and US to assess tumor-directed delivery, anti-tumor efficacy, and toxicity. They reported that anti-PD-L1-USMB inhibited tumor growth and concentrated ICI in tumor tissues to the greatest extent, evident by IHC staining, compared to control treatments consisting of anti-PD-L1-conjugated MB without US and free anti-PD-L1. Notably, IV injection of free anti-PD-L1 mAb resulted in significantly higher mortality (9/20 of tumor-bearing mice) than IV injection of anti-PD-L1-conjugated MB (2/20 of tumor-bearing mice), demonstrating that USMB improved tolerability [242]. Given that PD-1/PD-L1 ICIs are often combined with other drugs such as chemotherapy in the clinic [72,243], different studies have evaluated whether USMB-mediated PD1-blockade could synergize with other therapeutics. Li et al. and Ma et al. found that USMB using MBs conjugated to anti-PD-L1 mAb and loaded with DTX (denoted as PDMs) or co-administered IP with cisplatin, respectively, enhanced internalization of both therapeutics, increased CD8^+^ T cell infiltration and inflammatory cytokine levels, and induced tumor regression in subcutaneous models of LLC lung [244] and U14 cervical cancer [245]. Li et al. also reported that their PDMs combined with US enhanced tumor regression, prolonged survival times, and increased body weights in orthotopic lung tumor-bearing mice, relative to saline, free DTX, unconjugated MB, free DTX + anti-PD-L1 mAb, and PDM only controls [244]. This is remarkable as utilizing US for the treatment of pulmonary diseases has traditionally been perceived as unfeasible since air scatters US waves, preventing its penetration through the lungs [231,246]. For this reason, endobronchial ultrasound (eBUS) has been employed as an alternative diagnostic and staging technique to the traditional US applied through the chest for lung cancer [247]. In the study by Li et al., it is plausible that the accumulation of fluid in the tumor interstitium due to vascular leakage and dysfunctional lymphatics [238] enabled US beams to penetrate the chest and tumor sites while leaving healthy, aerated lung tissue unaffected. Indeed, in other models of pulmonary disease such as acute lung injury and acute respiratory distress syndrome, which are characterized by pulmonary edema, USMB was shown to enhance the uptake of therapeutic cargo specifically in diseased lung regions [248,249]. Given that eBUS is commonly used in the management of lung cancer patients, USMB may be a tractable strategy for focusing the delivery of drugs such as ICIs preferentially to lung tumors.

Liu et al. also demonstrated that USMB could be employed for combination therapy to deliver both an anti-PD-L1 mAb and synthetic *miR-34a* [250], a tumor-suppressive microRNA often downregulated in cancer [251]. *miR-34a* was attached to the cationic MB shell by electrostatic interactions, and the anti-PD-L1 mAb was conjugated by biotinylation. These decorated MBs preferentially targeted subcutaneous U14 cervical tumors upon US irradiation via direct binding of the anti-PD-L1 mAb to PD-L1 expressed on the surface of the cancer cells, which likely facilitated collateral uptake of *miR-34a*. This enhanced tumor killing and decreased tumor burden compared to treatment with unmodified MBs, anti-PD-L1-MBs, and *miR-34a*-MBs administered without US by activating apoptosis, evidenced by IHC and terminal deoxynucleotidyl transferase-mediated dUTP nick end labeling (TUNEL) immunostains. The combined anti-tumor effect was attributed to increased CD8^+^ T cell infiltration, downregulation of the anti-apoptotic protein Bcl2, and concurrent upregulation of the pro-apoptotic Bax protein [250]. These studies provide proof of concept that USMB can be leveraged to enhance the uptake and efficacy of PD-1/PD-L1 ICIs given as monotherapy or in combination with other therapeutics including chemotherapy and gene therapies.

As an alternative to conjugating MBs with ICIs and applying tumor-focused US, applications using USMB with co-administered free PD1/PDL1 ICIs are also being explored. Although this strategy is unlikely to reduce irAEs because the mAb is administered systemically, it may facilitate ICI penetration in specific tumor types by overcoming barriers that limit drug uptake. Brain malignancies are notoriously difficult to target with drugs [252]. The blood-brain barrier (BBB) is a semi-permeable filtering system between the blood and the brain. It poses a significant biological barrier to any therapeutics in circulation intended for the brain as it strictly limits the transport of molecules to protect it [253]. Therefore, some of the latest studies have focused on employing USMB to transiently perforate the BBB and deliver immunotherapeutics including PD-1/PD-L1 ICIs to brain tumors. A study orchestrated by Ye et al. investigated whether focused USMB could be used to enhance the delivery of free (i.e., unconjugated) fluorescently labeled anti-PD-L1 mAb to GL261 gliomas in the brainstem of tumor-bearing mice. Image-guided US was applied to the brainstem after the MBs were administered IV and the mAb intranasally for direct nose-to-brain contact. Ex vivo imaging coupled with immunofluorescence (IF) analysis of tumors from treated mice showed that focused USMB increased ICI uptake by 3.74-fold relative to controls treated with intranasal mAb only [254]. Focused USMB was also found to enhance brain-targeted delivery of IV-administered and fluorescently labeled anti-PD-L1 mAb in insonated regions of the brain compared to the contralateral non-insonated region in pigs [255]. While this was not a cancer study, Fadera et al.’s results are promising, since porcine models are more physiologically similar to humans than considerably smaller, anatomically and physiologically distant rodent models. A functional study by Ahmed et al. provided convincing data supporting USMB-mediated ICI efficacy in a syngeneic GL261 glioma model [256]. They demonstrated successful opening of the BBB by MRI and fluorescence imaging, and delivery of IP-injected anti-PD-L1 mAb to brain tumors by IHC. They also observed tumor regression and improved overall survival in the USMB + anti-PD-L1 group compared to the non-insonated (i.e., no US treatment) and free anti-PD-L1-treated controls [256]. These studies indicate the effectiveness of USMB in boosting the delivery of PD-1/PD-L1 ICIs to brain tumors which is tremendously challenging.

### 7.3. Challenges with USMB-Mediated ICI Delivery

While it has many advantages, USMB is also susceptible to delivery obstacles that cannot be overlooked. To begin with, MBs have a relatively short half-life in circulation, generally between three to five minutes, as a result of spontaneous degradation and clearance by the RE system [257,258]. The short half-life complicates its use as an ICI carrier vehicle because this significantly limits the amount of MBs that can penetrate the tumor, especially for those that are hypovascularized with few vessels supplying the tumor. Solutions to this issue are being explored; for example, MB surface modifications such as phospholipid coating have been shown to extend their half-life in vivo [107].

One of the most prominent issues preventing the clinical translation of USMB as a delivery method for ICIs is the lack of standardized delivery parameters [259] including mechanical indices (MIs), acoustic pressure, timing of treatment, and treatment intervals [260]. This is partly a reflection of the acoustic and physiological heterogeneity of the tumors, as well as the organs they are situated in: some organs and tumors are less accessible with US such as primary bone malignancies including osteosarcoma because of their acoustic properties [261,262]. In addition, many preclinical trials have experimented with customized MB formulations, for instance, cationic MBs for gene delivery [263], which adds further complexity and variability across studies, as changes in MB formulation can significantly alter their acoustic properties. As such, customized MBs require specialized US parameters. Since tailoring US machines and USMB parameters to patients with various malignancies in the clinic could be challenging, standardized MBs and US instruments may be required to translate this promising drug delivery technique. It is worth noting that the development of USMB for drug delivery is in its infancy relative to LDD, OVs, and NPs, as research in this field only started intensifying in the early 2000s [264]. Thus, it is arguably the most underdeveloped delivery method we have discussed, which may extend the time required to generate sufficient preclinical evidence to support its clinical translation.

## 8. Summary and Perspective

Cancer is studied intensely by researchers around the world who share the goal of discovering innovative treatments to combat this deadly disease. Due to the evolving nature of cancer and the molecular heterogeneity observed across and within individual tumors, patients respond differently to standard-of-care therapies, including immunotherapies such as ICIs. Differential responses to widely used PD-1/PD-L1 ICIs are seen with respect to their anti-tumor efficacy as well as the toxicities they cause in patients [265,266]. Therefore, continued research to overcome treatment obstacles associated with ICIs, such as drug resistance and irAEs, is essential for expanding their abilities to benefit patients. The clinically approved route and mode of administering ICIs using IV infusion of PD-1 or PD-L1-targeted mAb, combined with the expression of PD-L1 outside of tumor tissues, makes them prone to causing systemic off-tumor toxicities including potentially lethal irAEs. As such, significant efforts have been made to develop and implement novel ICI drug delivery strategies that can minimize their unwanted, dose-limiting side effects.

Here, we have described four alternative approaches for delivering ICIs targeting the PD-1/PD-L1 inhibitory immune checkpoint that is hijacked by many cancers to inhibit T cell-mediated tumor destruction. These strategies include the use of tumor-directed locoregional drug delivery (LDD), oncolytic viruses (OVs), nanoparticles (NPs), and ultrasound and microbubbles (USMB). We summarized a number of recent proof of concept studies that illustrate different variations of these modes of ICI delivery, which have all demonstrated effective delivery of PD-1/PD-L1 ICIs and associated therapeutic effects in diverse tumor types via distinct mechanisms. Treatment regimens not only included ICI administration as monotherapy but also as dual and even triple-therapeutic combinations, which elicited additive or synergistic anti-tumor effects. Most of these studies consistently reported enhanced tumor regression when the novel ICI delivery approach was tested alone and more pronounced tumor growth inhibition and survival benefits when combined with other therapies. Many studies also linked these anti-tumor phenotypes to the activation of adaptive immunity evidenced by an increase in tumor-infiltrating CD8^+^ T cells and upregulation of cytokines secreted by activated T cells within the TME, indicating successful PD-1/PD-L1 blockade. Encouragingly, a few studies noted abscopal regression of secondary tumors and rejection of cancer cells upon rechallenge indicating that these delivery methods were capable of inducing long-lasting immunological memory as a result of achieving effective PD-1/PD-L1 blockade. Moreover, anti-tumor efficacy was demonstrated using a variety of therapeutic agents including standard mAb but also siRNA, shRNA, antisense, DNA, small molecular inhibitors, and antagonistic peptides targeting the PD-1/PD-L1 signaling axis.

While these strategies to enhance tumor-directed ICI delivery are exciting, several outstanding questions must be addressed before they can progress to clinical trials in patients. First, while there are several reasons to develop novel ICI delivery strategies for cancer patients (e.g., cost of mAb, challenges with penetration of mAb into solid tumor tissues, suboptimal therapeutic efficacy in some patients), one of the most compelling is to minimize the risk of dose-limiting toxicities and potentially lethal irAEs. Although each of the studies we described reported anti-tumor efficacy, relatively few conducted thorough examinations to determine whether the alternative modes of ICI delivery caused adverse side effects in animal models. Additionally, few performed head-to-head comparisons of the anti-tumor efficacy and toxicities induced by the new delivery strategies to those by conventional IV infusion of mAb. Perhaps this is because most of these studies were proof of concept studies that focused on delivery and efficacy as primary outcomes rather than mitigation of irAEs caused by IV administration of ICIs. Nevertheless, studies that contrast conventional IV delivery methods with the alternative delivery methods we have discussed are imperative to inform their relative benefits with respect to enhancing ICI efficacy and reducing the incidence of irAEs. Furthermore, while several studies included readouts to confirm that these new modes of ICI delivery effectively inhibited PD-1/PD-L1 signaling and/or expression in tumors, few assessed the deposition of the therapeutic agents and their effects on PD-1/PD-L1 signaling/expression in non-malignant tissues. As such, studies to characterize the biodistribution of the drugs and their biological effects are also essential to prove that they do indeed focus delivery of ICIs to tumor tissues. Therefore, large preclinical trials in animal models are needed to robustly answer these questions before progressing these tumor-targeted ICI delivery strategies on the path toward clinical translation. Notwithstanding, given the promising results of these proof of principle studies, the precedent for using components of these delivery strategies in the clinic (e.g., US and MB for diagnostic imaging; T-VEC OV for melanoma; and DaunoXome NPs for chemotherapy delivery), and the fact that some of these strategies are already being investigated in clinical trials, we are optimistic that refinement of these techniques will result in their clinical approval to minimize irAEs induced by PD-1/PD-L1 ICIs. Achieving this feat will help to maximize the therapeutic potential of ICIs in the clinic by expanding their ability to benefit a broader population of patients.

## Figures and Tables

**Table 1:** Summary of the four alternative delivery methods.

Delivery Method	Concept	Specific Components	Evidence of Efficacy in Studies Described	Challenges
LDD	Direct injection of PD-1/PD-L1 ICIs into the tumor or placement of slow-release depots intra- or peri-tumorally for extended infusion of ICIs to concentrate ICIs within tumor tissue.	Hypodermic needles or microneedles Drug depots	Successful deposition of ICI in tumor tissue and TdLNs Enhancement of ICI therapeutic efficacy by sustained release into tumor tissue Increase in CD8^+^ T cell infiltration and inflammatory cytokine levels Inhibition of tumor growth Prolongation of survival times Compatible with allografts Absence of toxicity and enhanced tolerability of ICI Effective use with other non-ICI therapeutics *	Difficulty achieving uniform distribution of ICI within tumor tissue Potential for human error during injection (e.g., leakage of ICI into surrounding tissue) Limited applicability (i.e., amenable for superficial, palpable tumors such as melanoma)
Oncolytic virus (OV)	Preferential infection and targeted replication of OVs in tumor cells create an immunologically ‘hot’ TME primed for PD-1/PD-L1 ICIs upon induction of immunogenic cell death (ICD). Genetically engineered OVs can also encode ICIs to be released locally in the TME upon tumor cell infection and lysis.	Native or genetically modified OV	Successful deposition of ICI in tumor tissue Increase in CD8^+^ and CD4^+^ T cell infiltration and inflammatory cytokine levels Inhibition of tumor growth Prolongation of survival times Absence of toxicity in higher mammals (i.e., monkeys) Induction of long-term immunological memory Effective use with other non-ICI therapeutics *	Limited bioavailability in tumors when administered IV due to rapid clearance by the RE system Mostly limited to IT administration Resistance to certain OV strains (e.g., intrinsic resistance mechanisms or resistance due to the presence of neutralizing antibodies) Hesitance by the public due to concerns regarding viral therapy
Nanoparticle (NP)	NPs are used as carriers to shield ICIs in circulation and liberate them preferentially at tumor sites by exploiting the Enhanced Permeability and Retention (EPR) effect/Active Transport and Retention (ATR) effect and/or employing tumor-targeting modifications.	NPs (e.g., conjugated to or encapsulating ICI with other structural modifications to enhance tumor-targeting or factors to alter the TME to increase uptake	Successful deposition of ICI in tumor tissue Increase in CD8^+^ and CD4^+^ T cell infiltration and inflammatory cytokine levels Inhibition of tumor growth Prolongation of survival times Absence of toxicity Effective use with other non-ICI therapeutics *	Existence of biological barriers that limit NP uptake (e.g., inefficient EPR effect, formation of protein corona, clearance of NPs by the reticuloendothelial (RE) system, and dense tumor stroma) NP-related toxicities (e.g., induction of an inflammatory response and production of reactive oxygen species)
Ultrasound and Microbubble (USMB)	IV administration of MBs used as ICI carriers or co-administered with ICIs followed by application of US to tumor tissues which cavitates MBs in the tumor vasculature, causing release and accumulation of ICIs preferentially within the tumor via sonoporation, rearrangement of tight junctions, endocytosis, and vessel distention/invagination.	US gel, MBs, and a US transducer	Concentrated deposition of ICI in tumor tissue Increase in CD8^+^ T cell infiltration and inflammatory cytokine levels Inhibition of tumor growth Prolongation of survival times Enhanced tolerability of ICI Effective use with other non-ICI therapeutics *	Short half-life of MBs Lack of standardized delivery parameters across studies Use of customized MBs tailor-made for a given study

Note: * The content of Table 1 is discussed with references to the literature in Section 4, Section 5, Section 6 and Section 7 below.
